# The Role of Some Chemokines from the CXC Subfamily in a Mouse Model of Diabetic Neuropathy

**DOI:** 10.1155/2015/750182

**Published:** 2015-02-19

**Authors:** Magdalena Zychowska, Ewelina Rojewska, Dominika Pilat, Joanna Mika

**Affiliations:** Institute of Pharmacology, Polish Academy of Sciences, 12 Smetna Street, 31-343 Krakow, Poland

## Abstract

The mechanism involved in the development of diabetic neuropathy is complex. Currently, it is thought that chemokines play an important role in this process. The aim of this study was to determine how the level of some chemokines from the CXC subfamily varies in diabetic neuropathy and how the chemokines affect nociceptive transmission. A single intraperitoneal (*i.p.*) injection of streptozotocin (STZ; 200 mg/kg) resulted in an increased plasma glucose. The development of allodynia and hyperalgesia was measured at day 7 after STZ administration. Using Antibody Array techniques, the increases in CXCL1 (KC), CXCL5 (LIX), CXCL9 (MIG), and CXCL12 (SDF-1) protein levels were detected in STZ-injected mice. No changes in CXCL11 (I-TAC) or CXCL13 (BLC) protein levels were observed. The single intrathecal (*i.t.*) administration of CXCL1, CXCL5, CXCL9, and CXCL12 (each in doses of 10, 100, and 500 ng/5 *μ*L) shows their pronociceptive properties as measured 1, 4, and 24 hours after injection using the tail-flick, von Frey, and cold plate tests. These findings indicate that the chemokines CXCL1, CXCL5, CXCL9, and CXCL12 are important in nociceptive transmission and may play a role in the development of diabetic neuropathy.

## 1. Introduction

Diabetic neuropathy occurs in nearly 50% of patients with diabetes mellitus [1]. The mechanisms involved in the development of diabetic neuropathic pain are poorly understood due to their complexity. Currently, much attention has been given to the role of immune factors in the development of neuropathic pain [2–4], and it seems that the chemokines may play an important role in this process [5, 6]. We have shown that, during streptozotocin-induced diabetic neuropathy, the level of some pronociceptive interleukins and proteins from the tumor necrosis factor family is upregulated [7], which suggests their role in diabetic neuropathic pain. However, the role of chemokines in these processes remains unclear.

Chemokines are low molecular weight members of the cytokine family. There are four defined classes of chemokines: CC, CXC, CX3C, and XC. Each of them exerts its biological effects through a G-protein coupled receptor [8]. Their main function as chemoattractants is to guide the migration of receptor cells to the place of chemokine secretion. They are second line proinflammatory factors and are induced by primary proinflammatory mediators such as tumor necrosis factor (TNF) [9]. Recent studies have shown that chemokines from the CXC subfamily are involved in pain processes. CXCL1, CXCL11, and CXCL13 were upregulated in a SNL pain model [9, 10]. Furthermore, spinal application of CXCL1 or CXCL12 elicited pain hypersensitivity [9, 11] similar to intraplantar CXCL5 injection [12]. The chemokine receptors are expressed not only on immune cells but also on microglial cells or astrocytes [5, 6, 13]. The activated microglia cells, resident macrophages of the central nervous system, play an important role in diabetic neuropathy [7, 14]. Therefore, it seems that the chemokines may be crucial in the development and maintenance of diabetic neuropathic pain.

The aim of our study was to verify how the expression and protein levels of chemokines from the CXC subfamily vary with diabetic neuropathy. Using qRT-PCR and the RayBio Antibody Array technique, we examined the mRNA and protein levels of CXCL1 (KC), CXCL5 (LIX), CXCL9 (MIG), CXCL11 (I-TAC), CXCL12 (SDF-1), and CXCL13 (BLC) in lumbar spinal cords of diabetic mice. We verified also the influence of intrathecal administration of CXCL1, CXCL5, CXCL9, and CXCL12 on nociceptive transmission in naïve mice.

## 2. Materials and Methods

### 2.1. Animals

Male Albino-Swiss mice (30–35 g) were housed in cages lined with sawdust under a standard 12/12 h light/dark cycle (lights on at 08:00 h) with food and water available ad libitum. The experiments were carried out according to the Institute's Animal Research Bioethics Committee (Cracow, Poland), in accordance with IASP rules [15] and the NIH Guide for Care and Use of Laboratory Animals.

### 2.2. Induction of Diabetes

The type 1 diabetes model was obtained by a single intraperitoneal (*i.p*.) injection of streptozotocin (STZ; 200 mg/kg; Sigma Aldrich, USA) prepared in 0.9% NaCl [7, 16, 17]. Age-matched nondiabetic mice were injected with 0.9% NaCl. The glucose concentration was measured in blood collected from the tail vein. Mice with serum glucose levels above 300 mg/dL were considered diabetic. The behavioral tests were conducted on day 7 after STZ injection, to reflect changes in the early stage of diabetes development.

### 2.3. Behavioral Tests

#### 2.3.1. Tactile Allodynia (von Frey Test)

Allodynia was measured using calibrated nylon monofilaments (Stoelting, USA). von Frey filaments of increasing strengths were applied to the plantar surface of the hind paw of each mouse sequentially (from 0.6 to 6 g) until the hind paw was withdrawn [7, 18].

#### 2.3.2. Thermal Hyperalgesia (Cold Plate Test)

Hyperalgesia was assessed using the cold plate test (Cold/Hot Plate Analgesia Meter, Columbus Instruments) as described in our previous papers [7, 19]. The mice were placed on the cold plate (at a temperature of 2°C), and the time until the hind paw was lifted was recorded. The cut-off latency was 30 s.

#### 2.3.3. Nociceptive Threshold (Tail-Flick Test)

The pain threshold to a thermal stimulus was assessed by tail-flick latency determined by a tail-flick analgesic meter (Analgesia Meter; Ugo Basile, Comerio, Italy). In this test, the beam of light was focused on the dorsal tail surface 1 cm from the tip of the tail. The baseline was determined at 3–4.5 s. The cut-off time was 9 s [7, 20].

#### 2.3.4. Measurement of Blood Glucose and Body Weight

Blood glucose concentrations and body weights were measured at day 7 of the experiment. The glucose concentration was measured using an Accu-Chek Active Glucometer.

### 2.4. Biochemical Tests

#### 2.4.1. Analysis of Gene Expression by qRT-PCR

The lumbar (L4–L6) region of the spinal cord was dissected after decapitation of naïve mice and mice after STZ-induced neuropathy on day 7 following STZ administration. The total RNA was extracted with TRIzol reagent (Invitrogen) as previously described [21]. The RNA concentration was measured using a NanoDrop ND-1000 Spectrometer (NanoDrop Technologies), and RNA quality was determined by chip-based capillary electrophoresis using an RNA 6000 Nano LabChip Kit and Agilent Bioanalyzer 2100 (Agilent) [22]. Reverse transcription was performed on 2 *μ*g of total RNA using Omniscript Reverse Transcriptase (Qiagen Inc.) at 37°C for 60 min. RT reactions were carried out in the presence of an RNAse inhibitor (rRNAsin, Promega) and oligo (dT16) primer (Qiagen Inc.). cDNA was diluted 1 : 10 with H_2_O, and, for each reaction, ~50 ng of cDNA synthesized from the total RNA template obtained from individual animals was used for quantitative real-time PCR (qRT-PCR) reactions. qRT-PCR was performed using Assay-On-Demand TaqMan probes (Applied Biosystems) and run on an iCycler device (BioRad, Hercules). The amplification efficiency for each assay was determined by running a standard dilution curve. The following TaqMan primers were used: Mm00446968_m1 (Hprt, mouse hypoxanthine guanine phosphoribosyl transferase); Mm04207460_m1 (Cxcl1); Mm00436451_g1 (Cxcl5); Mm00434946_m1 (Cxcl9); Mm00444662_m1 (Cxcl11); Mm00445553_m1 (Cxcl12); and Mm04214185_s1 (Cxcl13). The expression of Hprt was measured and was quantified to control for variations in cDNA amounts. Hprt did not significantly change for STZ-injected mice and therefore served as a housekeeping gene (data not shown). The cycle threshold values were calculated automatically by iCycler IQ 3.0 software using default parameters. The RNA abundance was calculated as 2^−(threshold  cycle⁡)^.

#### 2.4.2. RayBio Mouse Inflammation Antibody Array

The lumbar (L4–L6) portion of the spinal cord was removed from naïve and STZ-induced neuropathic pain mice on day 7 following STZ administration. The tissue samples were homogenized in 1x Cell Lysis Buffer (RayBio) with Protease Inhibitor Cocktail (Sigma) and cleared by centrifugation (14000 ×g for 30 min). The protein concentration in the supernatant was determined using the BCA Protein Assay Kit (Sigma). The samples were diluted with 1x Blocking Buffer to a final concentration of 250 *μ*g of protein per sample.

The RayBio membranes (Table 1) were blocked at room temperature and incubated with 1 mL of sample overnight at 4°C. After incubation, the samples were decanted, and the membranes were washed 3x with 2 mL of 1x Wash Buffer I (RayBio) and 2x with 2 mL of 1x Wash Buffer II (RayBio) at room temperature. To each membrane, 1 mL of diluted Biotin-Conjugated Anti-Cytokines Antibodies (RayBio) was added and incubated at room temperature for 90 min. Then, the primary antibodies were decanted and the membranes were washed. The membranes were incubated for 2 hours at room temperature with 2 mL of 1000-fold diluted HRP-conjugated streptavidin (RayBio). The HRP-conjugated streptavidin was decanted, and the membranes were washed. Immunocomplexes were detected using Detection Buffer (RayBio) and visualized using a Fujifilm LAS-4000 fluorimager system. The relative levels of immunoreactivity were quantified using Fujifilm Image Gauge software [7].

### 2.5. Pharmacological Study

#### 2.5.1. Intrathecal Administration of Chemokines

The intrathecal (*i.t.*) administration was performed according to Hylden and Wilcox [23]. Using a Hamilton syringe with a thin needle, 5 *μ*L of each chemokine was injected between the L5-L6 vertebrae in the lumbar portion of the spinal cord. The tail reflex is an indication of the proper administration of the drug.

#### 2.5.2. Drug Administration

The chemokines were dissolved in water for injection. CXCL1/KC and CXCL5/LIX were obtained from Sigma Aldrich and CXCL9/MIG and CXCL12/SDF-1 were from R&D System. Reconstituted chemokines were injected in the following concentrations: 10 ng/5 *μ*L, 100 ng/5 *μ*L, and 500 ng/5 *μ*L. The behavioral tests were performed after 1, 4, and 24 hours following chemokine administration.

### 2.6. Data Analysis

The behavioral data for diabetic neuropathic pain development are presented as a percentage of the naïve mice ± S.E.M. of 4–16 animals per group. The results of the experiments were evaluated using Student's *t*-test. ^*^ *P* < 0.05 and ^***^ *P* < 0.001 indicate a significant difference versus naïve animals. For the chemokine administration, the data are presented as fold change of the control (naïve) ± S.E.M. The results were evaluated using a one-way analysis of variance (ANOVA). The intergroup differences were analyzed by ANOVA plus Bonferroni's Multiple Comparison Test; ^*^ *P* < 0.05, ^**^ *P* < 0.01, and ^***^ *P* < 0.001 indicate a significant difference compared with naïve animals. The data from the biochemical experiments are presented as fold change of the control (naïve) ± S.E.M. The qRT-PCR analysis represents normalized averages derived from the threshold cycle in qPCR from 4–10 samples for each group. The protein analysis was performed using the antibody array method from 11-12 samples per group. The results were evaluated using Student's *t*-test. ^*^ *P* < 0.05, ^**^ *P* < 0.01, and ^***^ *P* < 0.001 indicate a significant difference in comparison to the control (naïve animals).

## 3. Results

### 3.1. Mouse Diabetic Neuropathy Model

**Figure 1:**
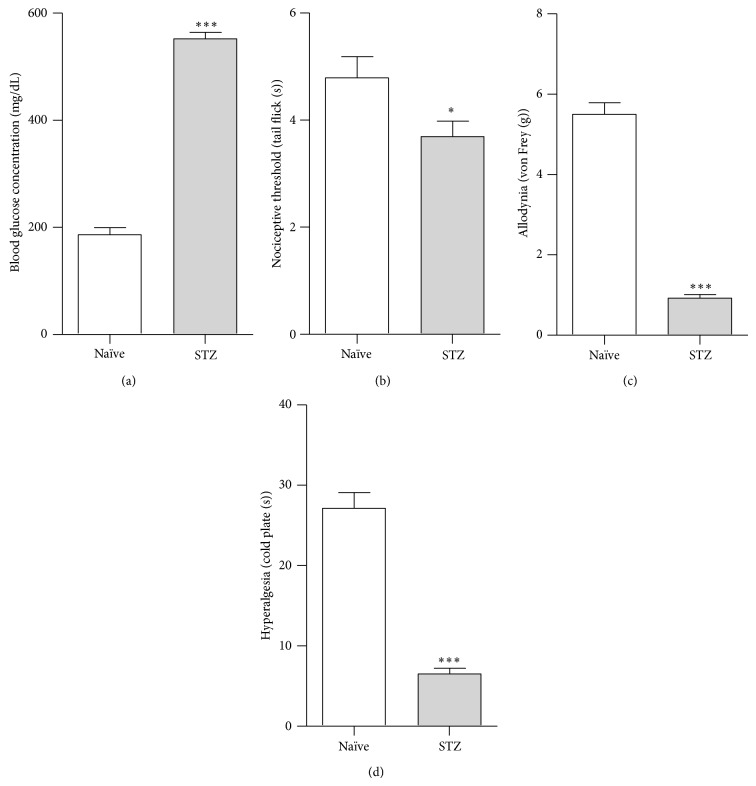
Mouse streptozotocin (STZ) model of diabetic neuropathy. Effects of single STZ (200 mg/kg;* i.p.*) administration on plasma glucose concentration (a); nociceptive threshold (tail-flick test) (b); allodynia (von Frey test) (c); and hyperalgesia (cold plate test) (d) measured at day 7. The data are presented as the mean ± S.E.M. (4–16 mice per group). The results were evaluated using Student's *t*-test. ^*^ *P* < 0.05 and ^***^ *P* < 0.001 indicate a significant difference versus naïve animals.

Seven days after STZ (200 mg/kg,* i.p*.) administration, an increase in the plasma glucose concentration in STZ-injected mice was observed (552 ± 12.0 mg/dL versus naïve 186 ± 13.6 mg/dL) (Figure 1(a)). Furthermore, at day 7 following STZ administration the decrease in nociceptive threshold, evaluated by the tail-flick test, was detected in STZ-injected mice (3.7 ± 0.3 s versus naïve 4.8 ± 0.3 s) (Figure 1(b)). Neuropathic pain syndromes such as allodynia and hyperalgesia were also detected at day 7 after STZ administration. The mechanical allodynia (0.9 ± 0.1 g) was assessed by the von Frey test (Figure 1(c)) and the thermal hyperalgesia (6.5 ± 0.7 s) was measured using the cold plate test (Figure 1(d)) in STZ-injected mice in comparison to naïve animals (5.5 ± 0.3 g and 27.2 ± 2.0 s, resp.).

### 3.2. qRT-PCR Analysis of Chemokines from the CXC Subfamily in a Diabetic Neuropathy Model

**Figure 2:**
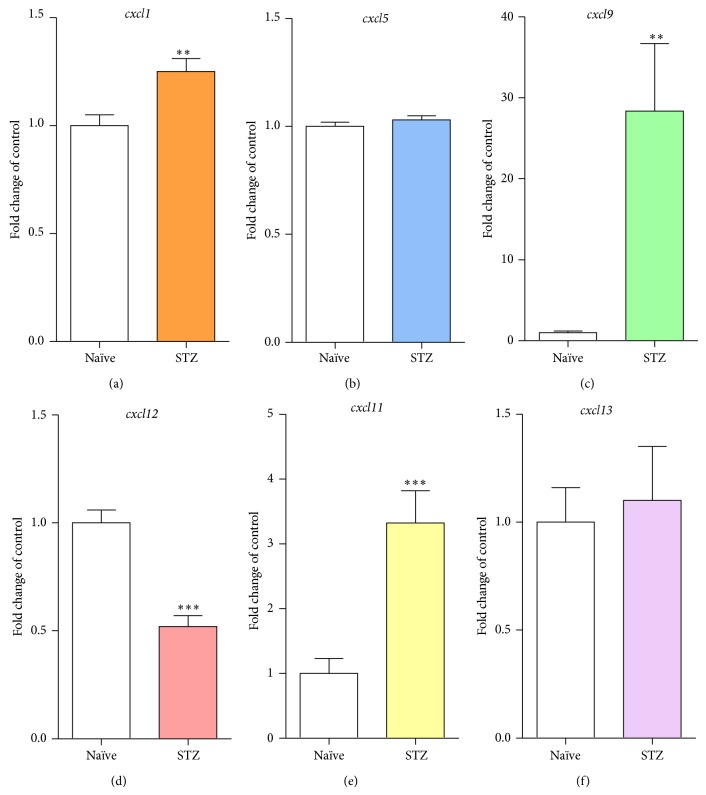
Changes in mRNA amount of chemokines from CXC subfamily after streptozotocin administration measured at day 7 in the mouse lumbar (L4–L6) part of the spinal cord. The qRT-PCR analysis of* cxcl1* (a),* cxcl5* (b),* cxcl9* (c),* cxcl11* (d),* cxcl12* (e), and* cxcl13* (f) expression in naïve and streptozotocin- (STZ-) treated animals. The data are presented as fold change in the control (naïve) ± S.E.M. (4–10 samples per group). The results were evaluated using Student's *t*-test. ^**^ *P* < 0.01 and ^***^ *P* < 0.001 indicate a significant difference compared with naïve mice. STZ, streptozotocin-induced diabetic neuropathy.

Seven days after* i.p*. STZ administration, the levels of* cxcl1*,* cxcl5*,* cxcl9*,* cxcl11*,* cxcl12*, and* cxcl13* mRNA were measured in the lumbar (L4–L6) portion of the spinal cord in mice using qRT-PCR (Figures 2(a), 2(b), 2(c), 2(d), 2(e), and 2(f)). In STZ-induced diabetic mice, a significant increase in* cxcl1* (Figure 2(a); 1.25-fold),* cxcl9* (Figure 2(c); 28.38-fold), and* cxcl11* (Figure 2(e); 3.32-fold) mRNA levels was detected. Simultaneously, a decrease in* cxcl12* (Figure 2(d); 0.48-fold) mRNA level and no changes in* cxcl5* (Figure 2(b)) and* cxcl13* (Figure 2(f)) levels in STZ-injected mice were observed.

### 3.3. Antibody Array Analysis of Chemokines from the CXC Subfamily in a Diabetic Neuropathy Model

**Figure 3:**
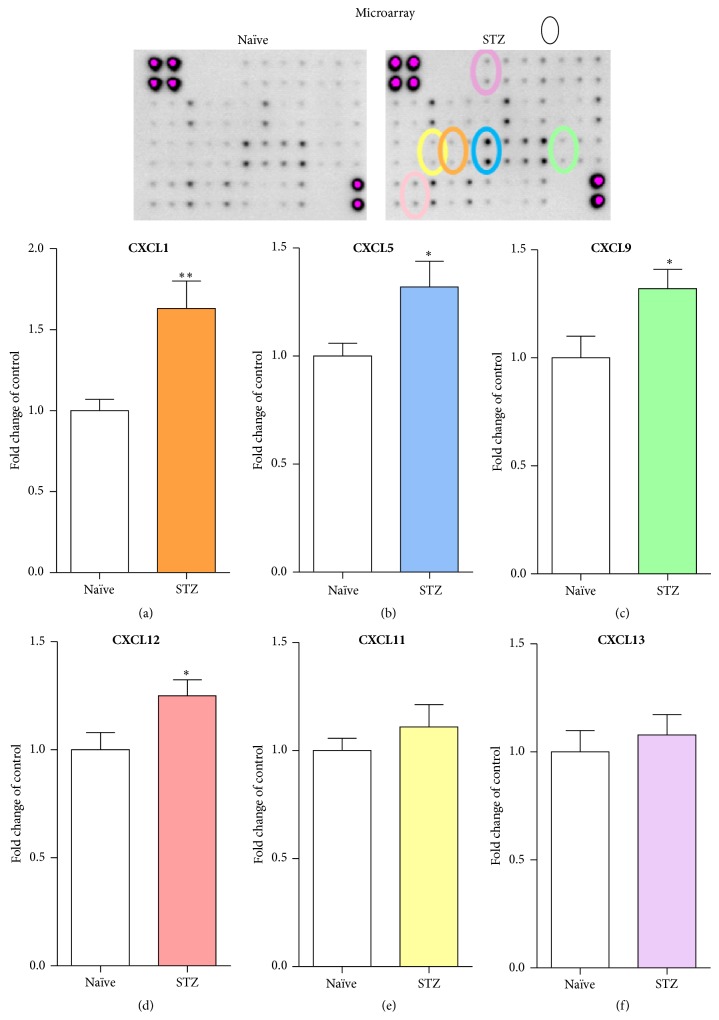
Streptozotocin- (STZ-) induced changes in chemokines from the CXC subfamily measured at day 7 in the mouse lumbar (L4–L6) portion of the spinal cord. The antibody array analysis of CXCL1 (a), CXCL5 (b), CXCL9 (c), CXCL11 (d), CXCL12 (e), and CXCL13 (f) protein level in naïve and STZ-treated animals. The data are presented as fold change in the control (naïve) ± S.E.M. (11-12 samples per group). The results were evaluated using Student's *t*-test. ^*^ *P* < 0.05 and ^**^ *P* < 0.01 indicate a significant difference compared with naïve mice. STZ, streptozotocin-induced diabetic neuropathy.

The analysis of CXCL1, CXCL5, CXCL9, CXCL11, CXCL12, and CXCL13 protein levels in the lumbar (L4–L6) portion of the spinal cord was conducted at day 7 after* i.p.* STZ administration, using the RayBio mouse inflammation antibody array (Figures 3(a), 3(b), 3(c), 3(d), 3(e), and 3(f)). In STZ-induced diabetic mice, an upregulation of CXCL1 (Figure 3(a); 1.63-fold), CXCL5 (Figure 3(b); 1.32-fold), CXCL9 (Figure 3(c); 1.32-fold), and CXCL12 (Figure 3(d); 1.25-fold) protein levels was observed. Changes in CXCL11 and CXCL13 levels were not detected (Figures 3(e) and 3(f)).

### 3.4. Effect of a Single Intrathecal Administration of CXCL1, CXCL5, CXCL9, or CXCL12 on Nociceptive Transmission in Naïve Mice

#### 3.4.1. Effect of a Single Intrathecal Administration of CXCL1, CXCL5, CXCL9, or CXCL12 on the Nociceptive Threshold Measured by a Tail-Flick Test in Naïve Mice

**Figure 4:**
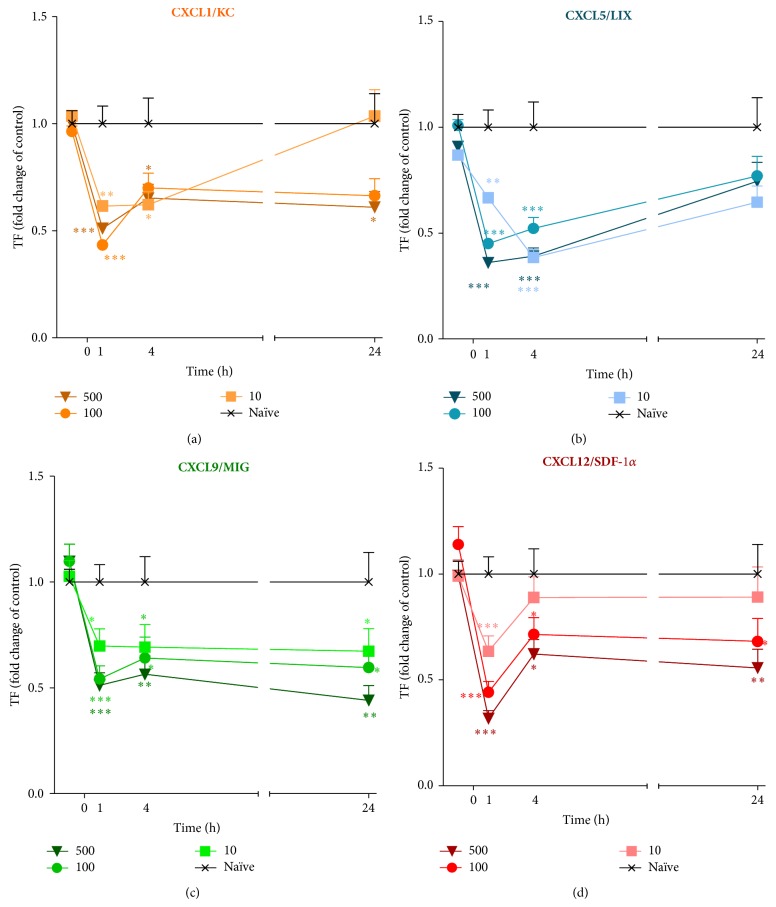
Effects of CXCL1, CXCL5, CXCL9, or CXCL12 administration on the nociceptive threshold. The influence of a single intrathecal administration of 10 ng, 100 ng, or 500 ng/5 *μ*L of CXCL1 (a), CXCL5 (b), CXCL9 (c), or CXCL12 (d) on the nociceptive threshold was evaluated using a tail-flick test. The measurements were taken 1, 4, and 24 hours after chemokine administration. The data are presented as fold change in the control (naïve) ± S.E.M. (6–16 mice per group). Intergroup differences were analyzed by an ANOVA and Bonferroni's Multiple Comparison Test. ^*^ *P* < 0.05, ^**^ *P* < 0.01, and ^***^ *P* < 0.001 indicate a significant difference; the statistical analysis was performed versus control (naïve) mice.

A single* i.t.* administration of CXCL1, CXCL5, CXCL9, or CXCL12 at each dose (10, 100, and 500 ng/5 *μ*L) caused a pronociceptive reaction as measured by the tail-flick test (Figures 4(a), 4(b), 4(c), and 4(d)). After CXCL1 (Figure 4(a)) treatment, the response at all doses to the thermal stimuli occurred in an hour after treatment and then slowly diminished. One day after injection, the values observed after the lowest dose (10 ng) were not significant in comparison to the control, but the highest dose (500 ng) still caused a pronociceptive reaction as measured by the tail-flick test (Figure 4(a)). The CXCL5 (Figure 4(b)) injection resulted in a dose-dependent response one hour after injection. Furthermore, at the later time points, 10 ng CXCL5 induced a reaction similar to that obtained with 500 ng of this chemokine (Figure 4(b)), and after 24 hours following the treatment the values were not statistically significant in comparison to the control (Figure 4(b)). One hour after CXCL9 application (Figure 4(c)), the 100 ng and 500 ng doses caused a similar response that was stronger than seen with the 10 ng dose. Furthermore, these effects were still detectable up to 24 hours later (Figure 4(c)). The CXCL12 (Figure 4(d)) injection resulted in a dose-dependent response one hour after injection. These effects were still measurable in the fourth hour after treatment for all doses except the 10 ng dose of CXCL12 (Figure 4(d)). Twenty-four hours following CXCL12 treatment, the pronociceptive properties of this chemokine were not observed in animals that received a dose of 10 ng (Figure 4(d)).

#### 3.4.2. Effect of a Single Intrathecal Administration of CXCL1, CXCL5, CXCL9, or CXCL12 on the Mechanical Allodynia as Measured by the von Frey Test in Naïve Mice

**Figure 5:**
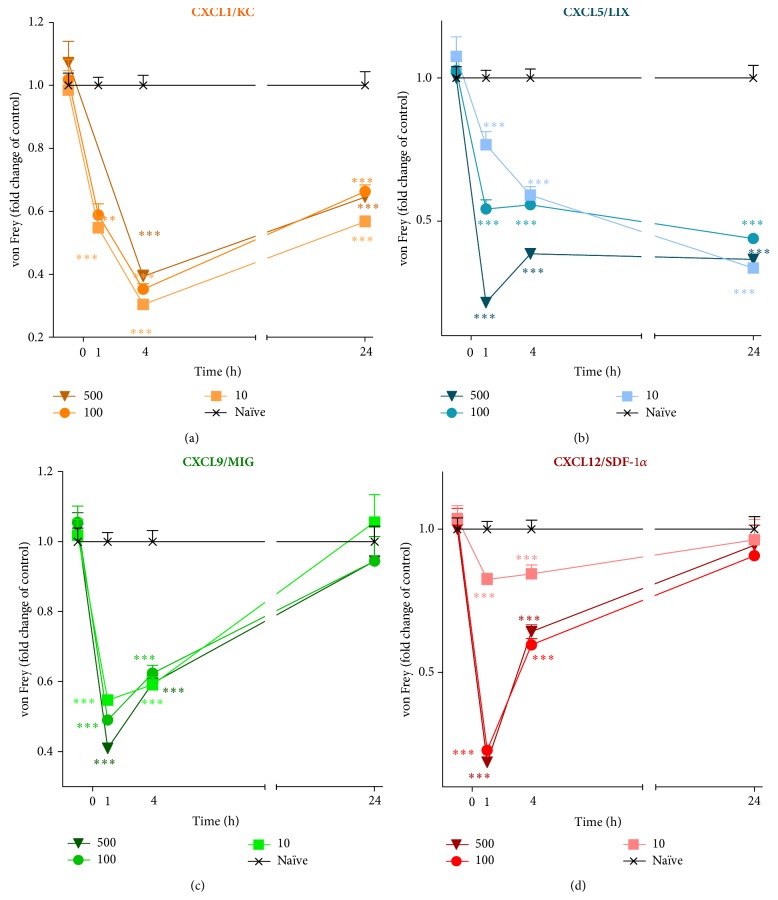
Effects of CXCL1, CXCL5, CXCL9, or CXCL12 administration on mechanical allodynia. The influence of a single intrathecal administration of 10 ng, 100 ng, or 500 ng/5 *μ*L of CXCL1 (a), CXCL5 (b), CXCL9 (c), or CXCL12 (d) on mechanical allodynia was evaluated using the von Frey test. The measurements were taken 1, 4, and 24 hours after chemokine administration. The data are presented as fold change in the control (naïve) ± S.E.M. (6–16 mice per group). Intergroup differences were analyzed by an ANOVA and Bonferroni's Multiple Comparison Test. ^*^ *P* < 0.05, ^**^ *P* < 0.01, and ^***^ *P* < 0.001 indicate a significant difference; the statistical analysis was performed versus control (naïve) mice.

A single* i.t.* administration of CXCL1, CXCL5, CXCL9, or CXCL12 at each dose (10, 100, and 500 ng/5 *μ*L) induced the development of mechanical allodynia as measured by the von Frey test (Figures 5(a), 5(b), 5(c), and 5(d)). One hour after CXCL1 treatment, a strong response to the mechanical stimuli was detected (Figure 5(a)). These effects were intensified in the fourth hour after treatment and were similar for all doses. Furthermore, 24 hours following administration, the mechanical allodynia was still observed (Figure 5(a)). CXCL5 (Figure 5(b)) or CXCL9 (Figure 5(c)) treatment resulted in a dose-dependent response in the von Frey test one hour following injection. Moreover, at the fourth hour after CXCL5 treatment, this correlation was still detected (Figure 5(b)) and persisted for up to 24 hours (Figure 5(b)). In the case of CXCL9 treatment, after four hours, all doses resulted in a similar, but weaker, reaction to mechanical stimuli than at one hour, which disappeared after 24 hours (Figure 5(c)). The administration of CXCL12 in doses of 100 ng and 500 ng caused a similar response in the von Frey test measured one hour after application (Figure 5(d)). Those effects gradually diminished in the fourth hour to achieve control values, measured one day after injection (Figure 5(d)).

#### 3.4.3. Effect of a Single Intrathecal Administration of CXCL1, CXCL5, CXCL9, or CXCL12 on Thermal Hyperalgesia as Measured by the Cold Plate Test in Naïve Mice

**Figure 6:**
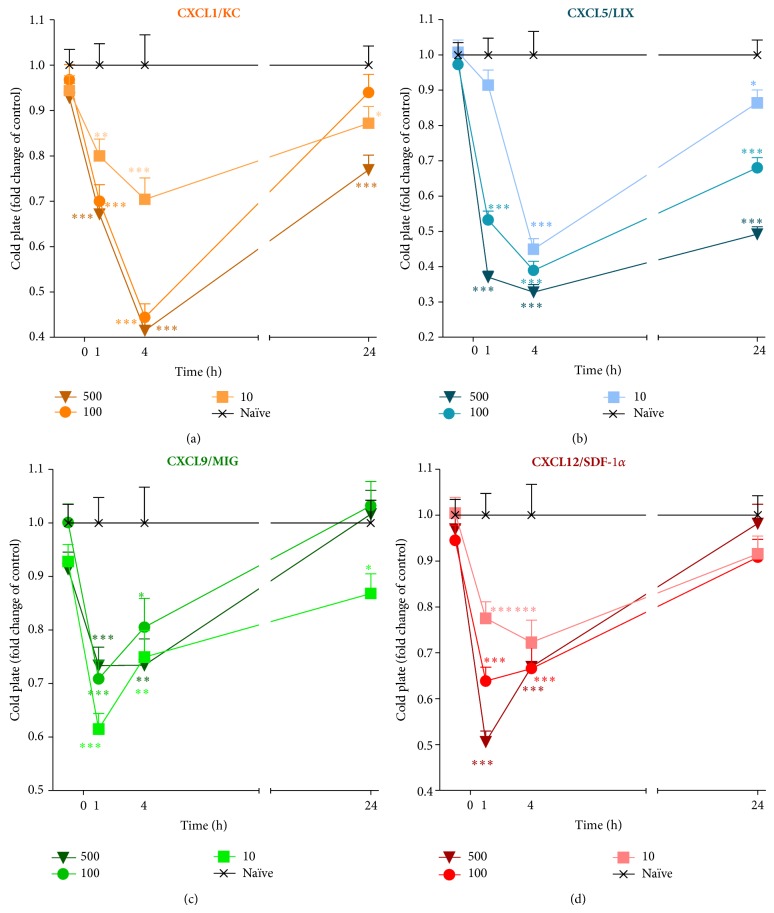
Effects of CXCL1, CXCL5, CXCL9, or CXCL12 administration on thermal hyperalgesia. The influence of a single intrathecal administration of 10 ng, 100 ng, or 500 ng/5 *μ*L of CXCL1 (a), CXCL5 (b), CXCL9 (c), or CXCL12 (d) on thermal hyperalgesia was evaluated using the cold plate test. The measurements were taken 1, 4, and 24 hours after chemokine administration. The data are presented as fold change in the control (naïve) ± S.E.M. (6–16 mice per group). Intergroup differences were analyzed by an ANOVA and Bonferroni's Multiple Comparison Test. ^*^ *P* < 0.05, ^**^ *P* < 0.01, and ^***^ *P* < 0.001 indicate a significant difference; the statistical analysis was performed versus control (naïve) mice.

A single* i.t.* administration of CXCL1, CXCL5, CXCL9, or CXCL12 at each dose (10, 100, or 500 ng/5 *μ*L) induced the development of thermal hyperalgesia as measured by the cold plate test (Figures 6(a), 6(b), 6(c), and 6(d)). One hour after CXCL1 treatment, a dose-dependent reaction to the thermal stimuli was evident for all doses (Figure 6(a)); by the fourth hour, those effects were potentiated. One day following administration of a 500 ng dose, CXCL1 still had strong pronociceptive properties (Figure 6(a)). In the case of CXCL5, the 100 ng and 500 ng doses triggered a dose-dependent pain response in the cold plate test at one hour after treatment (Figure 6(b)). In the fourth hour, the lowest dose (10 ng) induced a response to the thermal stimuli, although it was not as strong as seen for the other doses. The thermal hyperalgesia dose-dependent effect was still measured one day after treatment (Figure 6(b)). The results obtained after CXCL9 treatment showed that the reaction to the thermal stimuli at one hour was the same for the 10 ng and 500 ng doses (Figure 6(c)). Those effects, excluding 500 ng CXCL9, gradually diminished, and after one day thermal hyperalgesia was detected in only the animals that received 10 ng (Figure 6(c)). The dose-dependent effects obtained after CXCL12 application indicated their pronociceptive properties (Figure 6(d)). Furthermore, after four hours, the 10 ng dose induced the strongest reaction in comparison to the reaction measured after one hour. Two other doses caused similar but weaker reactions to thermal stimuli. After 24 hours, those effects were abolished (Figure 6(d)).

## 4. Discussion

This study verifies that, in streptozotocin-induced diabetic neuropathy, the spinal protein levels of CXCL1, CXCL5, CXCL9, and CXCL12, but not CXCL11 and CXCL13, are upregulated. Furthermore, the results show that the intrathecal administration of the chemokines that are changed in the spinal cord in STZ-induced diabetic mice (CXCL1, CXCL5, CXCL9, and CXCL12) are associated with the development of mechanical allodynia and thermal hyperalgesia in parallel to a decrease in the nociceptive threshold. Studying the neuropathic pain of diabetes using experimental diabetic animal models was demonstrated in 2012 by Wattiez et al. [24]. In the case of type 1 diabetes, the most common mouse model of chemoinduced pancreatic toxicity is streptozotocin-induced diabetes [16, 17, 25]. In our studies, a single dose of STZ induced the development of diabetes, as demonstrated by a high blood glucose level, and the development of neuropathic pain syndromes such allodynia and hyperalgesia, in agreement with our earlier experiments in a diabetic neuropathic pain model [7]. Furthermore, we observed that mice which developed diabetic neuropathy demonstrated a decrease in nociceptive threshold. Those results correlate with Ohsawa et al., who showed that up to four weeks the reaction to heat stimuli in the tail-flick test was quicker in diabetic mice than in naïve controls [25]; however, after 6 weeks the tail-flick latency was prolonged in diabetic mice which can be related to the progressive degeneration of the nerves within the tail of the animal [25].

Neuropathic pain induced by diabetes may be a neuroinflammatory disorder because it is mediated by a variety of inflammatory mediators [26, 27]. Recent studies have shown that, in STZ-induced diabetic neuropathy, some pronociceptive factors, such as IFN-gamma, IL-1 beta, and IL-6, are upregulated [7, 28]. However the participation of chemokines from the CXC subfamily in STZ-induced diabetic neuropathy remains unexplored. Currently, studies in many pain-related models suggest that chemokines from the CXC subfamily play an important role. The results of our studies suggest an important role for CXCL1, CXCL5, CXCL9, and CXCL12 in STZ-induced diabetic neuropathy.

In our experiments, the protein and mRNA levels of CXCL1, also known as keratinocyte-derived chemokine (KC) or growth-related oncogene (GRO), were increased in diabetic animals, and its intrathecal administration caused the development of neuropathic pain syndromes. Those results are in agreement with Zhang et al. [9], who observed the upregulation of CXCL1 expression in the spinal cord in the SNL model of neuropathic pain. Furthermore, the spinal injection of CXCL1 induces dose-dependent thermal hyperalgesia, while SNL-induced neuropathic pain was diminished by CXCL1 neutralizing antibody [9]. It was also reported that, in patients with diabetic neuropathy, the serum level of CXCL1 is higher than in healthy people [29]. In addition, CXCL1 seems to be crucial not only in neuropathic pain but also in inflammatory pain [30] and bone cancer pain [31]. In multiple sclerosis, the upregulation of CXCL1 was induced by proinflammatory cytokines such as IL-1 beta and IFN-gamma [32] and those cytokines were also increased in a streptozotocin-induced diabetic neuropathy model [7, 28].

Our studies also reveal an increase in the CXCL5 protein level; however, changes in the mRNA amount in the spinal cord were undetectable. Those discrepancies in mRNA and protein level can be related to the fact that process of translation does not always take place after transcription, but for some factors it can be shifted in time. Additionally, when the concentration of ligand is high and completely saturated its receptor the process of transcription is suspended; however translation may be still in process. In 2013, Liu et al. reported mechanical allodynia and parallel upregulation of CXCL5 mRNA in spinal lumbar dorsal horns after intrathecal injection of lipopolysaccharide [33]. Our results are in partial agreement with Nunemaker et al., who reported an increased serum level of CXCL5 in type 2 diabetics [34]. Furthermore, in UVB irradiation pain, CXCL5 causes mechanical pain-related hypersensitivity [12]. These reports confirm our experiments, in which the intrathecal application of CXCL5 induced the development of mechanical allodynia.

The changes observed in our study in the mRNA and protein levels of CXCL9 during diabetic neuropathy suggest its very important role in the neuropathic pain that develops in diabetes. CXCL9, also known as monokine-induced by gamma interferon (MIG), is a member of the IFN-gamma inducible subset of CXC chemokines. In our previous studies, we have shown that IFN-gamma is upregulated in the spinal cord 7 days after STZ treatment [7], which may explain the strong parallel upregulation of CXCL9. A number of previous studies have suggested a role for the cytokine IFN-gamma within the dorsal horn in chronic pain states [35]. Therefore, CXCL9 as a target of IFN-gamma signaling may be involved. Our results obtained after intrathecal CXCL9 administration confirm this hypothesis. We have observed that, after injection of this chemokine, the nociceptive threshold was lower and neuropathic pain syndromes developed. Additionally, after stimulation of Schwann cells with high glucose levels, CXCL9 mRNA expression was elevated and high levels of CXCL9 protein were detected in the supernatant [36]. Moreover, the expression of CXCL9 was increased in the joints of patients with rheumatoid arthritis [37] and it was found that activation of spinal CXCL9 mediated bone cancer pain in rats [38]. A pronociceptive role for CXCL9 within the dorsal horn would provide further support for the use of chemokine antagonists in the treatment of diabetic neuropathic pain.

CXCL12, formerly named stromal cell-derived factor 1 (SDF-1), is expressed in various kinds of cells in the central nervous system. It has been shown that increased signaling by SDF-1/CXCR4 contributes to chronic pain behavior [27, 39]. Therefore, the results obtained in our studies showing an upregulation of CXCL12 in the lumbar spinal cord and the decrease in its mRNA content in diabetic animals correlate with this thesis. As was mentioned previously, this inconsistency may be associated with a shift in the process of transcription and translation. In STZ-induced diabetic neuropathy, SDF-1 increased the velocity of sciatic nerve conduction [40], and in spinal cord injury the CXCL12 was induced in the dorsal horn after spinal cord lesioning [41]. Moreover, some authors have observed an increase in CXCL12 in the spinal cord in bone cancer pain [39] and in antiretroviral toxic neuropathy [42]. Furthermore, spinal administration of CXCL12 to naïve mice caused the development of allodynia and hyperalgesia. Others have shown that intrathecal administration of CXCL12 induced the development of mechanical allodynia [11]. Moreover, single spinal administration of CXCL12 neutralizing antibody reversed the pain hypersensitivity induced by tumor cell implantation [39].

Recent studies demonstrate the participation of CXCL11 and CXCL13 in different pain states. Therefore we examined the role of these chemokines in STZ-induced diabetic neuropathy. CXCL11 is called interferon-inducible T-cell alpha chemoattractant (I-TAC) and interferon-gamma-inducible protein 9 (IP-9). Gene expression of CXCL11 is strongly induced by IFN-*γ*, and it is chemotactic for activated T cells. We observed in spinal cord tissue an increase in CXCL11 mRNA level. These findings corresponded to McColl et al. [43] who indicated the increase of CXCL11 mRNA expression in experimental autoimmune encephalomyelitis (EAE) in spinal cord. However, in our study, the spinal protein level of this chemokine in STZ-diabetic mice was not changed which may be associated with a shift in the process of transcription and translation. We also did not observe differences in spinal CXCL13 protein and mRNA level between naïve and STZ-treated mice. However, in animals with EAE, but not in naïve controls, the CXCL13 mRNA was detected. Furthermore, CXCL13-deficient mice exhibited a mild, self-limited form of this disease [44]. It has also been suggested that CXCL13 could be a diagnostic marker of Lyme neuroborreliosis [45] and rheumatoid arthritis [46]. Our results suggest that, at day 7, CXCL11 and CXCL13 are not involved in STZ-induced diabetic neuropathy.

In conclusion, the results of our study suggest that increased spinal levels of CXCL1, CXCL5, CXCL9, or CXCL12 protein in mice after STZ treatment may participate in the development of diabetic neuropathic pain. Furthermore, we have shown that intrathecal administration of CXCL1 and CXCL5 to naive mice caused immediate development of allodynia and hyperalgesia, but those effects were still observed 24 hours following administration. Because diabetic neuropathy is a neuroinflammatory disorder, investigations should focus on better understanding the role of chemokines in its development and maintenance. A more accurate explanation of the participation of these chemokines in diabetic neuropathy has been planned for future studies.

## Figures and Tables

**Table 1:** We used RayBio Mouse Inflammation Antibody Array 1 (40).

		A	B	C	D	E	F	G	H	I	J	K	L
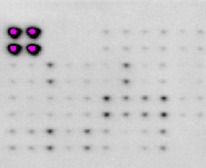	1	POS	POS	NEG	NEG	Blank	**BLC**	CD30 L	Eotaxin	Eotaxin-2	Fas Ligand	Fractalkine	GCSF
	2	POS	POS	NEG	NEG	Blank	**BLC**	CD30 L	Eotaxin	Eotaxin-2	Fas Ligand	Fractalkine	GCSF
	3	GM-CSF	IFN*γ*	IL-1*α*	IL-1 *β*	IL-2	IL-3	IL-4	IL-6	IL-9	IL-10	IL-12p40p70	IL-12p70
	4	GM-CSF	IFN*γ*	IL-1 *α*	IL-1 *β*	IL-2	IL-3	IL-4	IL-6	IL-9	IL-10	IL-12p40p70	IL-12p70
	5	IL-13	IL-17	**I-TAC **	**KC**	Leptin	**LIX**	Lymphotactin	MCP-1	MCSF	**MIG**	MIP-1*α*	MIP-1*γ*
	6	IL-13	IL-17	**I-TAC**	**KC**	Leptin	**LIX**	Lymphotactin	MCP-1	MCSF	**MIG**	MIP-1*α*	MIP-1*γ*
	7	RANTES	**SDF-1**	TCA-3	TECK	TIMP-1	TIMP-2	TNF*α*	sTNF RI	sTNF R II	Blank	Blank	POS
	8	RANTES	**SDF-1**	TCA-3	TECK	TIMP-1	TIMP-2	TNF*α*	sTNF RI	sTNF R II	Blank	Blank	POS
